# Pulmonary placental transmogrification: a difficult pattern in differential diagnosis of pulmonary hamartomas from a tertiary care hospital in Turkey

**DOI:** 10.1186/s13019-023-02217-1

**Published:** 2023-04-11

**Authors:** Busra Yaprak Bayrak, Cigdem Vural, Kursat Yildiz

**Affiliations:** Department of Pathology, Faculty of Medicine, Kocaeli University, 41380 Kocaeli, Turkey

**Keywords:** Placentoid bullous, Placental transmogrification, Pulmonary hamartoma

## Abstract

**Objective:**

Pulmonary placental transmogrification (PT) is a benign lesion curable by resection, represented by an unusual peculiar morphological variation including placentoid bullous change in the pulmonary hamartoma. In this retrospective study, we aimed to examine the histopathological features of pulmonary hamartomas in lung, to evaluate the different histological components, especially PT, and to investigate importance of PT pattern and its relationship with other clinicopathological features.

**Methods:**

Thirty-five cases of pulmonary hamartomas were recruited from the records between 2001 and 2021, divided into two groups according to presence of PT, as PT (-) and PT (+) in pathological examination.

**Results:**

77.1% of all patients were male. There was no significant difference between the two groups in terms of age, sex, comorbidity, presence of symptoms, tumor localization, and radiological findings (P > 0.05). Pulmonary hamartomas were resected totally from 28 patients (80%). Five of these patients (17.9%) had PT components in resection materials with varying degree between 5 and 80%, and all were from male patients. Examination with frozen sections were performed in 15 PT (-) and 5 PT (+) patients but diagnosis with frozen sections was not achieved in any of PT (+) patients. Most of materials included chondroid components (52.22 ± 29.7%) in both groups (P < 0.05).

**Conclusion:**

The placental papillary projections are available patterns associated with a pulmonary hamartoma and these projections observed especially in frozen sections are very crucial to recognize PT pattern in hamartomas, as they can result in confusions in differential diagnosis of malignities.

## Introduction

The definition of hamartoma is “a focal tissue malformation resembling a neoplasm caused by maldevelopment of the organ” [1]. The most common benign tumor of the lung are pulmonary hamartomas mostly observed as peripheral solitary foci consisting of the cartilage, fat, mesenchymal tissue and occasional bronchial glands, which are components of normal lung tissue. As a third most common cause of solitary pulmonary nodules, the pulmonary hamartoma typically grows at a similar rate to normal tissue components and, unlike true neoplasms, rarely cause compression of normal adjacent tissue [2, 3]. Pulmonary hamartomas are detected incidentally on routine radiological examinations such as radiography and computed tomography (CT). Radiographic diagnostic findings include calcification or an adipose component [4].

Pulmonary placental transmogrification (PT), also called placentoid bullous change, is a benign lesion curable by resection, represented by an unusual peculiar morphological variation in pulmonary hamartoma [5]. Morphologically, PTs demonstrate a microscopic resemblance to immature placental tissue in terms of the papillary projections lined by cuboidal or ciliated columnar cells, although they do not carry any biological and biochemical features of a placenta [6]. The presentation of PT ranges from asymptomatic to clinically overt and PTs are related to the pulmonary diseases including repeated pneumothoraxes, chronic obstructive airway disease, centrilobular emphysema with bullous features, organized bronchopneumonia, and even respiratory distress [7, 8]. Referring to approximately 15% of cases, PTs are infrequently incidental lesions observed by radiography [5]. The hamartomas without PT lesion are easily diagnosed; however, some rare cystic tumors, such as congenital lesions (e.g., adenomatoid cystic malformation), alveolar adenoma and sclerosing hemangioma challenge the differential diagnosis of PTs [9]. The source and pathogenesis of PT are unidentified, however, there are hypotheses of pathogenesis claiming a lymphatic or vascular proliferation in emphysematous lung parenchyma [10], a component of a congenital malformation or an increase in mast cells in the stroma of hamartomas [11] but none of these hypotheses are based on sound morphologic or biological evidence.

Until now, approximately 50 reported cases of pulmonary PT in the English literature [11–23]. To our best knowledge, there is no study reporting that PT was histopathologically diagnosed in a hamartoma, and presented more than one case in a single center. In this retrospective study, we aimed to examine the histopathological features of pulmonary hamartomas in the lung, to evaluate the different histological components of these hamartomas, especially PT, and to investigate the importance of the PT pattern and its relationship with other clinicopathological features.

## Methods

### Patients

A total of 35 cases of pulmonary hamartomas diagnosed at the Department of Pathology, XXX University Faculty of Medicine, from 2001 to 2021 were selected for the study. The patients’ age, sex, comorbidities and symptoms, the localization and radiological findings of hamartomas, the types of biopsies, the application of frozen sections were recorded. All patients were divided into two groups according to the presence of PT, as PT (-) and PT (+).

All procedures performed in studies involving human participants were in accordance with the principles of Declaration of Helsinki. A written informed consent was obtained from all individual participants included in the study. Ethical consent was obtained from the local ethics committee for this study.

### Cytopathological and histopathological examination

The fine needle aspirations cytology (FNAC) was performed by using a 25-gauge needle attached to a 10-ml syringe under the guidance of CT. The number of passes was dependent on the size of the nodule and the amount of material obtained with each pass. Three to four passes were done in the majority of cases. The smears were air-dried and stained without fixation using May-Grünwald-Giemsa staining or fixed by alcohol and stained by Papanicolaou. The number of smears ranged from one to 10 with an average of four slides per case.

All pulmonary tissues were fixed in 10% neutralised formaldehyde. The resected tissues were cut into 3-mm thick slices to view the nodules. Materials were totally embedded in paraffin, cut into 5-µm sections then stained with haematoxylin and eosin (H&E).

The examinations of all surgically excised lesions followed the compliance standards and were performed under light microscope by two specialist pathologists. Hematoxylin-eosin–stained slides of 30 pulmonary hamartoma cases were examined in terms of hamartoma components, percentages of these components, presence of villous papillary projections or placenta-like structures^11^ and the ratio of these structures to the tumor.

### Statistical analysis

GraphPad version 3.06 2003 software was used for statistical analyses. Kolmogorov-Smirnov test was used to test the normality. Two independent continuous variables normally distributed were compared with unpaired t test with Welch correction while those not normally distributed with Mann-Whitney Test. Two categorical variables were compared with Chi-square Test and three or more categorical variables were compared with Chi-squared Test for Independence. A P-value less than 0.05 was considered significant.

## Results

### Demographical, clinical and radiological findings

**Fig. 1:**
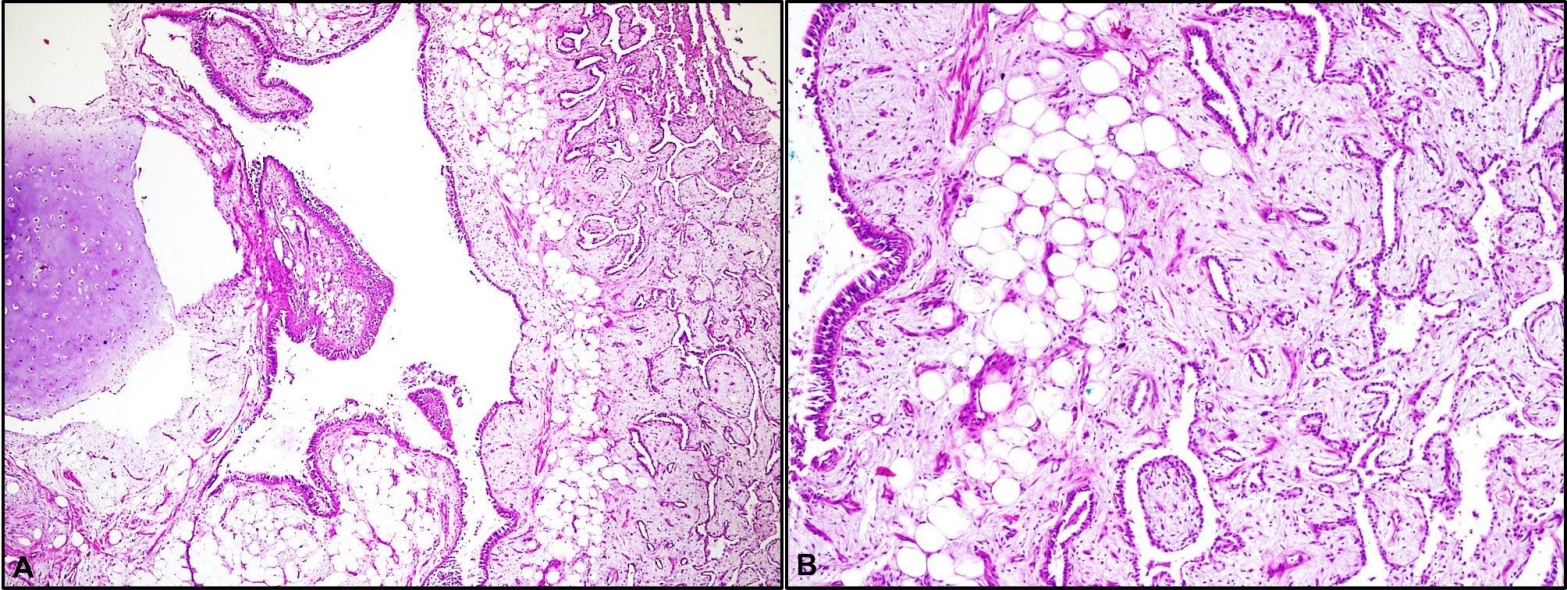
Morphological features of placental transmogrification in pulmonary hamartoma. (A) Myxoid and edematous stroma (H&E, x40). (B) Magnified micrograph of A (H&E, x100)

Thirty-five cases of pulmonary hamartoma resected by tru-cut biopsy or resection including the wedge resection, bronchotomy, segmentectomy and lobectomy were identified from 20 year- departmental records. The patients’ age ranged from 33 to 79 years, with a mean of 60.1 ± 14.1 years. Most of the patients (77.1%) were male. Five (14.3%) male patients were found to have a well-developed pattern of PT (Fig. 1). The mean age and distribution of sex did not differ between the PT (-) and PT (+) groups (p > 0.05) (Table 1). The sizes of pulmonary hamartomas ranged from 0.3 to 3.7 cm (median: 2 cm). Most of patients were asymptomatic (n = 21), and 12 patients in PT (-) group (40%) and two patients in PT (+) group (40%) were found to be symptomatic in the clinical examination (Table 1). All of the symptoms were mild respiratory symptoms including chest pain and cough.

**Table 1:** Demographical, clinical and radiological features of patients with pulmonary hamartoma

	Total (n = 35)	PT (-) (n = 30)	PT (+) (n = 5)	P value
Age, Mean ± SD	60.1 ± 14.1	59.6 ± 14.1	63.2 ± 2.8	0.5144
Sex, N (%)				
Male	27 (77.1)	22 (73.3)	5 (100)	0.4596
Female	8 (22.9)	8 (26.7)	0 (0)	
Comorbidities, N (%)				
Leiomyoma	3 (8.6)	3 (10)	0 (0)	0.7922
Pulmonary malignities	5 (14.3)	4 (13.3)	1 (20)	
Other malignities	4 (11.4)	3 (10)	1 (20)	
Symptoms, N (%)				
Symptomatic	14 (40)	12 (40)	2 (40)	1.00
Asymptomatic	21 (60)	18 (60)	3 (60)	
Localization, N (%)				
Central	4 (11.4)	4 (13.3)	0 (0)	0.9136
Peripheral	31 (85.6)	26 (86.7)	5 (100)	
Radiology Findings, N(%)	29 (82.9)	24 (80)	5 (100)	0.6471

PT: Placental transmogrification

Chi-squared Test or Chi-squared Test for Independence

Evaluation of comorbid diseases indicated that three patients in PT (-) group had leiomyoma, one had squamous cell carcinoma in the same lung, three had adenocarcinoma in the same lung, one had synovial sarcoma in one leg, one had multiple myeloma, and another had rectal carcinoma. In PT (+) group, one patient had pulmonary adenocarcinoma in the other lung and another had prostate carcinoma. The distribution of comorbidities was not significant among patients with or without PT (p = 0.7922) (Table 1).

In radiological examination, the hamartomas of PT (-) group were mostly located peripherally (86.7%), while the lesions were peripheral in all patients of PT (+) group, however, there was no significant difference between the groups (p = 0.9136). The distribution of pulmonary hamartomas did not present any lobar predilection. 82.9% of patients showed a radiological finding including calcification and fat density and there was no significant difference for radiological finding between the patients with or without PT (p = 0.6471) (Table 1).

### Pathologic findings

Of all patients, FNAC procedure was applied on seven patients (20%) and all smears were acellular and non-diagnostic. A hamartoma diagnosis was set in these seven patients by tru-cut biopsy simultaneously performed by aspiration. No PT was detected in FNAC and tru-cut biopsy samples of these patients.

Tumors of the rest of patients (n = 28) were resected totally. In 5 of these patients (17.9%), PTs were detected in the resection material. In 17 of these patients, frozen samples were collected and 12 were diagnosed with hamartoma but not with PT. The patients in PT (+) group whose frozen samples were collected were not diagnosed for malignity. Their differential diagnosis was set after evaluation of paraffinized sections.

In all cases of PT (+) group, a frozen sampling was needed while 50% of 30 PT (-) cases needed frozen sampling. There was a significant difference for the diagnosis with frozen sectioning between two groups (P = 0.0478) (Table 2).

**Table 2:** Histopathological features of the pulmonary hamartomas

	Total (n = 35)	PT (-) (n = 30)	PT (+) (n = 5)	P value
Diagnosis with Trucut	*N (%)*	*N (%)*	*N (%*	0.546
Diagnosis with	7 (20)	7 (23.3)	0 (0)	
Resection	28 (80)	23 (76.7)	5 (100)	
Examination with Frozen section	20 (57.1)	15 (50)	5 (100)	0.0478
Hamartoma				
Components (%)				
Chondroid	52.22 ± 29.70	61.5 ± 27.99	31.0 ± 27.25	*0.0328*
Epithelial	9.38 ± 4.86	8.57 ± 5.07	8.33 ± 2.89	0.8408
Fibromixoid	17.5 ± 25.45	24.21 ± 27.29	22.5 ± 17.56	0.9353
Adiposis	15.63 ± 17.08	27.22 ± 17.34	13.75 ± 11.82	0.1152
Ossification	5.0 ± 12.39	17.5 ± 12.55	5	*-*
Smooth muscle	95	95	-	*-*

PT: Placental transmogrification

The hamartomas were usually 4 cm or less, sharply delineated and lobulated, unencapsulated. The appearances of tumors were glistening, translucent, homogenous, tan-white to gray without cystic change or hemorrhage. The cut surfaces were firm to hard (cartilaginous) with ill-defined clefts and connective tissue septa. Two cases showed gross papillary projections within hamartomas, with a granular cut surface.

**Fig. 2:**
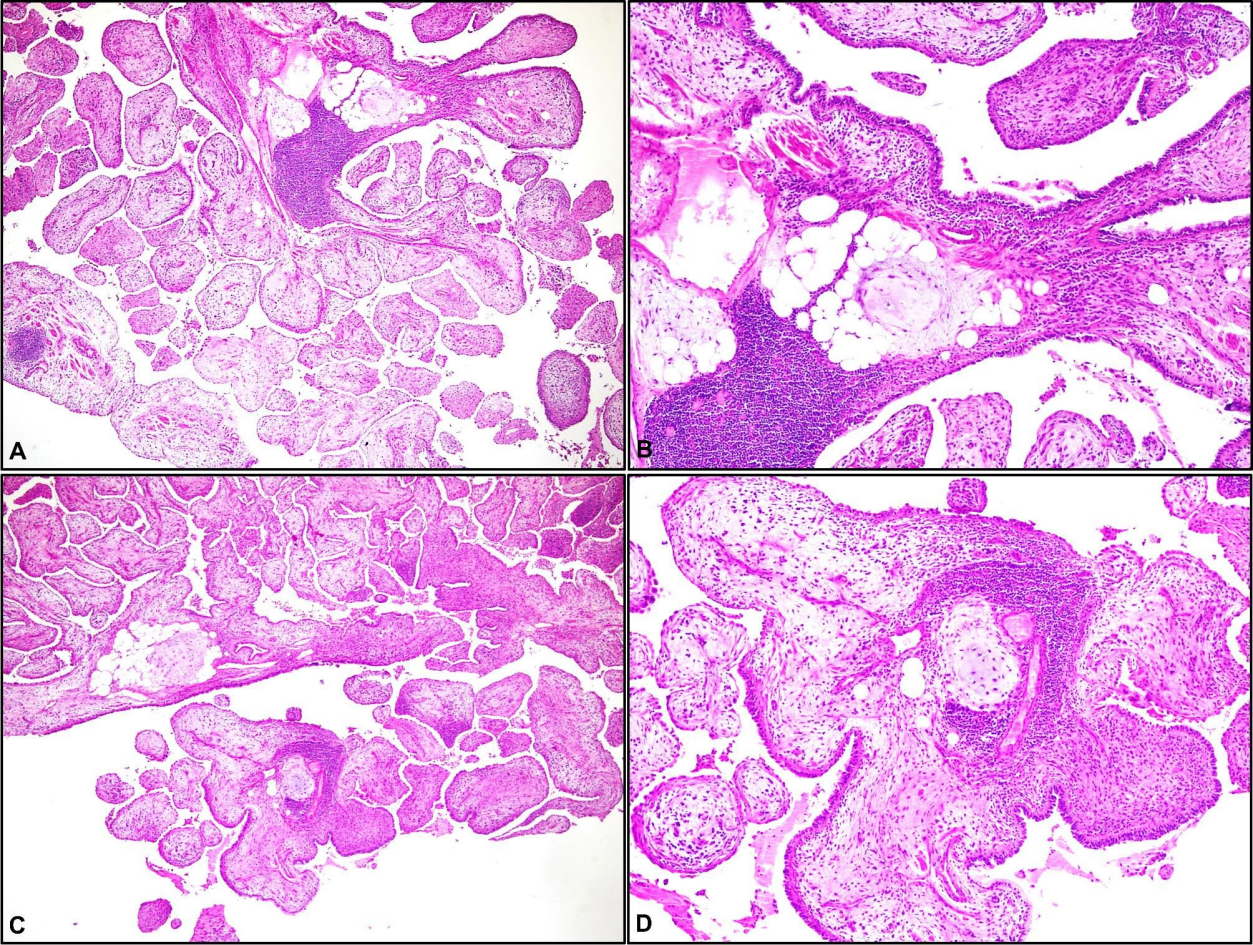
A. Papillary projections with adjacent area of a lymphoid aggregate (H&E, x40). B. Magnified micrograph of A (H&E, x100). C. Villus-like projections with cartilage and lymphoid aggregates (H&E, x40). D. Magnified micrograph of C (H&E, x100)

**Fig. 3:**
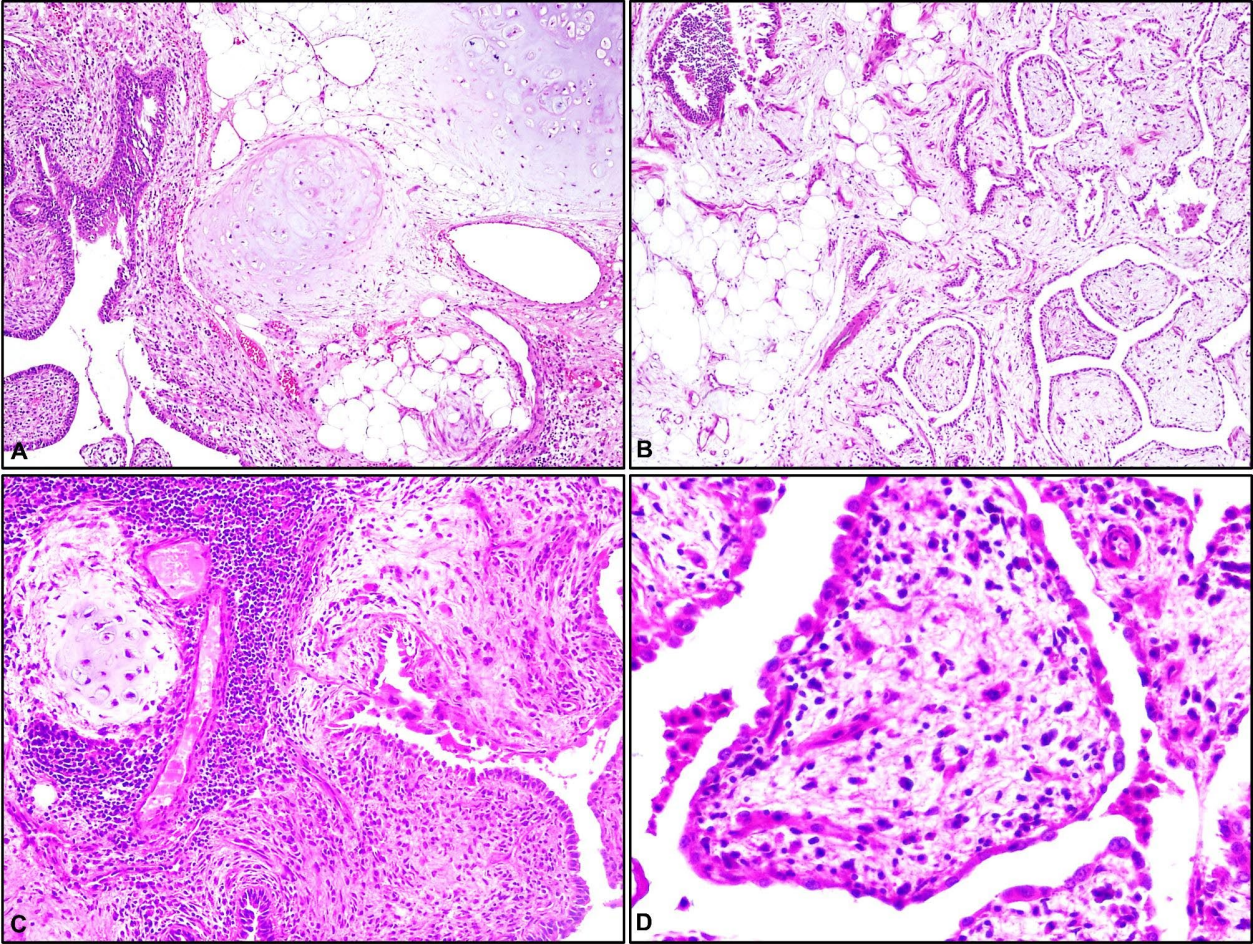
A. The stroma of pulmonary hamartomas with PT contained fibroadipose tissue, blood vessels and many inflammatory cells including the lymphocytes and macrophage, all diffusely dispersed (H&E, x100). B. Prominent epithelial foldings formed the papillary projections and myxoid stroma with vessels in villus-like structure (H&E, x100). C. Epithelial and chondroid components with dense lymphocytic inflammation (H&E, x100). D. Non-ciliated and ciliated columnar epithelial lining of immature placental villi (H&E, x200)

Microscopically, the hamartomas consisted of chondroid tissue, fibromixoid stroma, adipose tissue, epithelial components, smooth muscle, varying proportions of ossification. The adjacent lung frequently presented a deposits of type II pneumocytes, intra-alveolar macrophages and slight interstitial fibrosis. Most of materials (n = 31) included chondroid components (52.22 ± 29.7%) in both groups and these components in PT (-) group were larger than those in PT (+) group (P = 0.0328) (Table 2). All cases presented varied amounts of myxoid or edematous stroma (Fig. 1A and B). In all cases with PT, prominent epithelial foldings formed the papillary projections, reminiscent of immature placental villi (Fig. 2A and D). The stroma of pulmonary hamartomas with PT contained fibroadipose tissue, blood vessels and many inflammatory cells including the lymphocytes and macrophage, all diffusely dispersed (Fig. 3A C). The lining epithelium consisted of non-ciliated and ciliated columnar cells (Fig. 3D). The hamartomas of patients with PT showed a varying degree of PT components between 5 and 80% and other degrees of chondroid, epithelial, fibromixoid and adiposis components, as presented in Table 3. 5% ossification was detected in only one hamartoma with PT (Table 3).

**Table 3:** Histopathological features of the pulmonary hamartomas with placental transmogrification

Patient No	PT (%)	Chondroid (%)	Epithelial (%)	Fibromixoid (%)	Adiposis (%)	Ossification (%)
1	5	70	10	10	5	0
2	60	40	0	0	0	0
3	20	5	5	40	30	0
4	5	35	10	35	15	0
5	80	5	0	5	5	5

## Discussion

Pulmonary hamartomas are usually small, solid, subpleural localized benign lung tumors which may contain PT components. Most of the pulmonary hamartomas which were identified incidentally contained a significant amount of cartilage tissue, constituting solid lesions in lung. At the histological level, both central and peripheral pulmonary hamartomas consisted of irregular proliferation of mature mesenchymal tissues. The differential diagnosis of these PTs from malignant tumors of the lung is essential [5, 6]. In the present study, we examined the histopathological features of pulmonary hamartomas in lung and evaluated the PT patterns whose differential diagnosis from the malignancies in pulmonary hamartomas were challenging especially in frozen sections. Pulmonary hamartomas were resected totally from 80% to 35 patients and 17.9% of these patients had PT components in resection materials with varying degree between 5 and 80%.

The periphery of cartilage in pulmonary hamartomas may contain immature myxomatous tissue, but if there is no cartilage, it resembles to breast fibroadenoma, lipoma or leiomyoma, hence the differential diagnosis of these diseases may be challenging histopathologically [1, 9, 11]. Therefore, these lesions without a chondroid component were not easily diagnosed as pulmonary hamartomas. In fact, if there is no chondroid component in tru-cut biopsies, it may be difficult to diagnose these hamartomas with other distinctive features and the patient may be referred to surgery. In the present study, most of hamartomas included chondroid components and these components in PT (-) group were larger than those in PT (+) group. All of our tru-cut biopsy cases had a chondroid component, suggesting that these patients do not need resection and the follow-up of the patients continue.

Transthoracic FNAC has become a popular and reliable method for the diagnosis of pulmonary mass lesions if the samples are properly prepared for the cytology, and a number of studies have reported that a differential diagnosis of pulmonary hamartoma is possible by FNAC in most cases [24, 25]. Cytologically, FNAC represents an identifiable mesenchymal component with either fibromyxoid or chondroid features, admixed with groups of bland columnar epithelial cells, and may indicate a myxofibromatous tissue that is metachromatically stained with Giemsa and Wright dyes, strongly suggesting that the lesion is a hamartoma [1]. In the present study, 7 patients underwent a FNAC procedure; however, all of these aspirations are acellular, and none of the patients had pulmonary hamartomas were diagnosed by FNAC but identified by Tru-cut biopsy. The reasons for this conclusion may be that the aspiration was not taken with the appropriate technique or the radiologist was inexperienced or the delivery of insufficient aspiration material.

Pulmonary hamartomas grow slowly and most are smaller than 4 cm, but can reach 10 cm in diameter [10] as reported in our study showing a size ranged between 0.3 and 3.7 cm, mostly located in the peripheral parenchyma of lung but rarely centrally. Since the lesions tend to grow at the same rate as the surrounding tissue, the hamartomas generally do not cause compression symptoms in the adjacent lung parenchyma [9].

Among patients with PT associated with hamartoma, the clinical presentation was similar to those without this component. In the literature, most of pulmonary hamartomas are fibrochondromatous type with a ratio ranging between 71 and 90% [11, 26]. The age of patients with PT diagnosis varied between 20 and 90 years and men predominate over women by a ratio of 2:1 [26]. These findings are consistent with those of our patients who had chondromatous type pulmonary hamartoma, with an age range of 33–79 years and all were male. However, there are controversial data for the age and sex distribution of cases with PTs [11].

Approximately 15% of pulmonary hamartomas demonstrate a microscopic resemblance to immature placental tissue. These hamartomas with PT component show varying degrees of development of papillary structures, often lined by a single layer of epithelial cells or with epithelial invagination into stroma. The epithelium covering the papillary projections may be flat, cuboidal, or columnar type, and some of the cells are ciliated [5, 11]. In our study, the rate of hamartomas containing PT component was similar to the literature (14.3%), and the epithelium of these structures consisted of ciliated or non-ciliated columnar cells. Morphologically, the origin of these cells is the respiratory epithelium or represents an extension from adjacent lung epithelium. There are reports showing the epithelium lining the placenta-like projections are mitotically active, suggesting that the greater proliferation of epithelial lining cells over relatively inactive stromal cells results in glandular inclusions or placental villus-like structures [6, 8, 11]. This increase in the epithelial clefting may explain the papillary pattern of PT. Although it was frequently noted in PT cases associated with bullous emphysema in the literature [12, 16, 17], any lymphatic or vascular channel formation and dilation were not observed in our sections. Therefore, the origin and pathogenesis of PT associated with the pulmonary hamartomas may differ from PT associated with bullous emphysema [9, 10].

Apart from the epithelial cells, any multinucleated cells, pleomorphism or necrosis are not observed in the pulmonary hamartomas [11], as in our cases. In addition, a metaplasia and hyperplasia may be observed in entrapped airway elements [27], but not in our cases.

When PT is detected in pulmonary hamartoma histopathologically, an intralobar pulmonary sequestration (IPS), congenital cystic adenomatoid malformation (CCAM), bronchogenic cysts and cystic lung carcinoma should be considered in the differential diagnosis. IPS is a solid mass of abnormal pulmonary tissue that does not communicate with the tracheobronchial tree and is supplied by an anomalous systemic artery [28]. CCAM is a cyst lined by ciliated columnar type epithelium, not pneumocytes, and uncommon among adults [29]. Bronchogenic cysts are closed sacs lined by ciliated epithelium, involving focal areas of hyaline cartilage, smooth muscle and bronchial glands within their walls. These cysts are considered to be the result of an abnormal budding of the respiratory system [30–32].

Lastly, the cystic lung carcinoma presents cystic airspaces preceded by nodules which can evolve into non-small cell lung carcinoma. This carcinoma may also show wall thickening or mural nodularity. Histologic sections and cytology smears of the carcinoma will show malignant tumor cells. In our study, in paraffin sections, all of the masses, including PT with hamartomas, were examined, and their epithelium was solid masses resembling bronchogenic cyst epithelium, containing chondroid or adipose elements. Therefore, the diagnosis was set easily. However, it was difficult to diagnose hamartomas with PT in frozen sections, especially when other accompanying components were not seen. Malignant lesions, especially papillary type adenocarcinoma, could not be completely excluded. The masses were removed by a wedge resection, and the diagnosis of pulmonary hamartoma could be accomplished in the sections examined after frozen procedure. Similarly, it is emphasized that it is difficult to distinguish benign-malignant lesions if suspicious pulmonary masses sent to frozen contain PT and care should be taken [32].

The most significant consideration when encountering a possible hamartomatous lesion should go to distinguishing the lesion from malignancies. There are two studies reported PT associated with lung adenocarcinoma but the relationship between PT and carcinoma cannot be elucidated [7, 31]. In our study, a pulmonary adenocarcinoma was detected in one of 5 patients diagnosed with PT but not located in the same lung with pulmonary hamartoma. It may be appropriate to remove and sample the pulmonary hamartoma masses completely because of this possibility of malignancy that may be detected with the benign lesions.

The pulmonary hamartomas with or without PTs are curative benign lesions which can be removed by surgery as an only definitive treatment option available. A minimal resection may be selected and a lobectomy may be avoided [33]. This approach of minimal resection, however, is dependent on extent of underlying bullous disease and size of hamartoma / nodule, and in cases where it is associated with malignancy and the extent of surgery is dictated by tumor stage [7, 31].

As for the treatment, the prognosis of pulmonary hamartomas depends on underlying associated disease progress [34, 35]. Pulmonary hamartoma recurrence was not detected during follow-up of our cases. Only 1 case died due to accompanying adenocarcinoma complications.

As a limitation of the present study, there was an uneven distribution of cases with and without pulmonary PT. The low number of cases with PT in hamartoma may cause a bias in statistical results. However, PT cases are rare as reported in the literature, and as a result of our 20-year retrospective analysis, we could only reach 5 cases among hamartomas. Such a long-term follow-up and investigation of the presence of PT in all hamartomas demonstrates the strength of our study.

## Conclusion

In conclusion, in our study in which we investigated the demographic, clinical and histopathological characteristics of hamartomas, we would like to emphasize that placental papillary projections are patterns associated with pulmonary hamartoma, that the diagnosis of these projections may not be possible in frozen sections, and this pattern that can be confused with malignancy should be kept in mind when there is no other hamartoma components such as fibrochondromatous. The origin of PTs may be the proliferating respiratory epithelium overlapping the stromal component of hamartoma. Upon exclusion of malignancy, the benign pulmonary hamartomas should be considered when deliberating the nature of solitary pulmonary nodules.

## Data Availability

All data generated or analyzed during this study are included in this published article.
